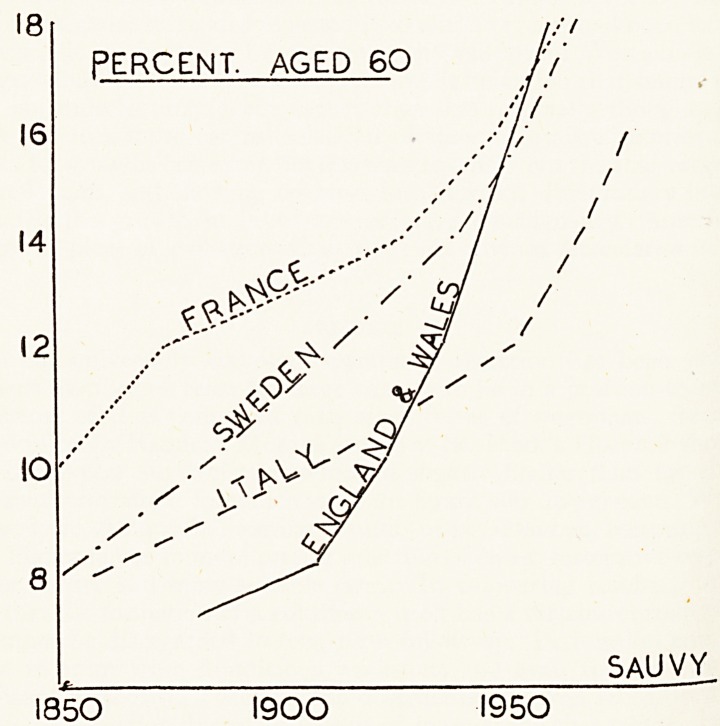# The Geriatric Service

**Published:** 1961-01

**Authors:** T. W. Lloyd


### THE GERIATRIC SERVICE

BY

T. W. LLOYD

It has become necessary to revise the medical and welfare services for the elderly because of the stark facts of change in the world's population. Whereas care for the elderly is not a serious problem in communities having no more than two or three per cent of old people, once that figure rises beyond a certain level the burden becomes a very different matter. Respect and reverence for the aged, such as is traditional in the Chinese and Indian cultures is hardly practicable when more than one person in twenty is sixty years old; and when the number approaches one in five, as it does in this country and indeed in all which have practised "Western" medicine and "Western" civilization, the burden may seem very heavy indeed. Just how the picture will vary in the future is difficult to forecast, for the pattern of the birth rate is unpredictable, but if we could now establish biological equilibrium, exactly replacing each dying generation by a new one, approximately 24 per cent of our people would be over sixty years old.

That we in Britain are not alone in this is shown by comparing the change in other European countries. In the figure the numbers over the past century in France, Italy and Sweden are compared with those for England and Wales, and each country is seen to be experiencing the same trend. Many people seem to suppose that the large increase in the number of old people about today is due to the effect of clinical medicine on the preservation of people already old, but life tables show that this change is due far more to increased expectation of life among young people than in the middle aged or elderly. In the U.S.A. life expectancy at birth has increased from 40 years in 1850 to 50 in 1900, 60 in 1930 and 70 today. Similar figures obtain in this country. In contrast to this dramatic increase of 20 years over half a century, the expectation of life of a man or woman already aged 60 has increased only two and a quarter years in the same period. This comparison shows that our increasing number of elderly people is due rather to the control of killing disease in youth, and perhaps in middle age, than to "medicated survival" of the elderly. It is indeed the case that the proportion of truly long-lived people, the nonagenarians, so many of whom remain vigorous to a remarkable degree, is no higher today than a hundred years ago.

What is the startling fact and the basis for a new look at old age is summed up in these figures: Great Britain had one million people over 64 years of age in 1851, five million in 1947 and is likely to have eight million by 1977. Every practitioner who had pre-war experience must have felt the impact of this change—that is why I have thought it necessary to examine the reason for the geriatric service before looking at the thing itself. I do not wish to seem sentimental about "the Old Folks"; the plain statistical fact is that there are far more of them than there used to be and they cannot be dealt with on the same basis as in the past.

This fact established, one must add that the treatment of the elderly sick does not need justification on statistical grounds. Old people need and deserve something better than the care they were offered under the old dual hospital system. Pressure on beds in acute medical and surgical wards led to the too early transfer of many old people to the chronic sick wards of the Poor Law Infirmary where the services needed to ensure maximum recovery were virtually never available. Unless these wards were exceptionally well staffed, they became too easily repositories in which those whose recovery was slow and problematical rapidly lost any chance they may have had. The reputation of such places was therefore not good and difficulties were aggravated by the reluctance of many families to permit their elderly sick to go to "the Workhouse Infirmary". In this desperate struggle many parents lost at the same time the affection of their children and their hope of rehabilitation.

It must be the first object of any geriatric service to provide Time as well as Care; time in which the patient can slowly regain strength and resources for a resumption of the burden of living, time and space in which everything possible can be done to prevent her becoming one of "The Chronic Sick". It is our function to open as wide a gap as possible between failing strength or acute illness and that final classification, and it is a little depressing sometimes to find how few doctors in general or consulting practice appreciate this function. To be asked to take a "Chronic Sick" patient as though we were but middlemen on the road to that final consumer, the undertaker, is not necessarily a stimulating experience.

What the Geriatric Service likes is to be offered the chance of maintaining the sufferer from permanent or progressive disabling disease in a state of maximum activity and happiness, preferably at home; to teach the patient how to live "within the limits of her disability but to the hilt of her capacity" (Rusk 1959). For this, early reference of the case is desirable, not a late and hopeless appeal for a "Chronic Sick" bed.

I have already stressed the need for time. Time alone, with intelligent nursing care, can result in remarkable recoveries. Early this year a woman of 92 came into my care following a myocardial infarct and pneumonia. She was utterly tired of life, so frail and feeble, so despairing that it seemed impossible to hope for recovery. Yet one day, 10 weeks after her illness began I noticed her chatting volubly with an old acquaintance and from that moment a rapid and complete recovery followed.

But time is seldom so effective of itself. Rather has it to be used effectively, first to correct chemical imbalance, circulatory failure, malnutrition, infection in urine or lungs, and later by positive re-training to educate the patient back to the fullest possible mobility. This can be done most effectively only if the patient is first admitted to a hospital unit fully equipped to bring modern medical facilities into play as they may be needed. One such unit per geriatric service will normally suffice and should contain 20 per cent of the total number of beds. Though roughly equal numbers for the two sexes may be necessary in the assessment unit, nearly three times as many long-stay beds will be needed for women as for men, fewer of whom suffer from chronic infirmity. I will not weary you with figures of beds available here except to let you who work in this area know that their distribution in small parcels in seven hospitals in the Gloucester and four in the Cheltenham group is awkward and inefficient. The great drawback to having many small places is that one cannot visit them often, so they can only be used as long-stay repositories in the main. In the interest of the nurses one must introduce some variety into the sort of case under their care; one must remind them that discharge from their hospital is a possibility; but it is not in the interest of the patients that anyone should go direct into a local long-stay hospital who might benefit from the stronger medical staffing which should prevail in the central assessment unit. It is a generally accepted belief in geriatrics that better results come from increasing the central unit until it reaches the optimal size rather than from adding beds to outlying long-stay hospitals. The essence of the modern method is to seek by early accurate diagnosis and effective treatment to limit the number of patients who require long-stay hospital care. At the same time we recognize that in rural communities the possession of conveniently placed long-stay hospitals is a great boon to families and friends.

The service throughout the country though very varied has other accepted general principles. One of these is that one individual should have responsibility for an area, in most cases a consultant physician. There has been no school of training for geriatrics but the field itself, and men from a remarkable diversity of professional backgrounds have made notably successful contributions to its practice. It is clear that what old people need is attention to their disease and a sympathetic appraisal of the possibility of their recovery, combined with the resources to exploit that possibility wherever it is found to exist.

### SOCIAL ASPECTS

I find the social background to geriatric medicine wholly fascinating. The different ways in which families react to the illness of parents continually bring surprise. The mutual tolerance which is established by some is a very different matter from the mutual dependence one sees in other relationships whether the partners are parent and daughter, sisters, or husband and wife. Illness may bring a disruptive or cohesive force to such relationships and it is important to know with which one is dealing. Prompt admission may be all-important in handling sickness in one case if the social unit is to have any chance of survival, yet in another admission to hospital may be genuinely dangerous to a social symbiosis which is built on mutual dependence. Often enough there may be some question as to whether the mother or the daughter, the dependent or the prop, stands most in need of treatment. Such situations are especially prone to develop when the illness of the mother coincides with the daughter's climacteric. In the presence of children embarrassment may become even more acute.

Many geriatric physicians feel that they cannot appraise the social situation at second hand, and make it a practice to visit all patients in their homes before admission. Others depute this matter to an Almoner or Local Authority Health Visitor, holding that to do the work themselves is an unprofitable waste of time. I use both methods and find great value in the visits I do myself for they give me an accurate picture of social unit of which my patient is a part.

I do not subscribe to the view that the bond of dutiful feeling between relations is much less strong than it used to be, but with the increasing numbers and proportion of elderly people one must recognize that this feeling needs support. Only if the relatives are able to resume the care of the patient can we achieve our object of restoring her to her place in society, and there certainly are many situations in which the onset of illness in the old person may be hailed as an opportunity to shrug off the burden of social strain for ever. Early contact between geriatric physician and family may put this matter in true perspective on both sides. While the physician will feel that his patient has social rights *vis-a-vis* the family that must be supported, and must consider the most profitable use of his beds in the service of the community, he must also recognize the implications and effect of the situation on the family or other social support. It is essential, especially in the three generation family, to see that the social rights of the disabled grandparent are not always given priority over those of the children and grandchildren.

Much use can and should be made of the geriatric beds for supporting the family. In my unit we run a limited service of this kind, some sixty men and women are being taken in during the summer months this year for periods of two or three weeks to enable the family to go on pre-arranged holidays. We have to be inflexible in these arrangements and it is often desirable that one of the hospital staff should see the relatives or visit the home in advance to decide whether any form of treatment can be attempted simultaneously with the custodial care, and whether a longer stay should be planned with this in view. Nevertheless from time to time the best laid plans go wrong. Isaacs and Thompson (1960) have reported that of forty-six such admissions only twenty-six patients returned home on time, six died, two went to Welfare Homes, twelve stayed longer than planned. I think this is too high a figure of failure and have had much better success myself, but one does in this way come across patients who can benefit from longer periods of treatment. We do of course give periods of relief at other times than the summer holiday if the indication is sufficient.

There are other forms of support which the hospital service can give. DeLargy (1957) believes in the value of regular, rhythmic relief to relatives, and has set aside a ward into which he admits patients for six weeks after intervals of six weeks; six weeks in, six weeks out. He reckons that thereby he gets two patients into each bed over the year yet sustains their feeling of social importance. He claims also that someone is always looking forward happily to something; either the mother to her return home or the daughter to the impending spell of relief! Few of us can emulate DeLargy exactly but recurrent admissions at more or less regular intervals may be the kind of support which will prevent a family from getting exhausted to the point at which they want at all costs to be rid of their incubus.

### DIAGNOSIS

It may seem impertinent to tell an audience of doctors that diagnosis is an important step toward sucessful treatment. I do so only because it has so often been allowed to go by default in chronic sick wards. In most new geriatric services examination of these people yields a rich harvest of remediable conditions and in many practices patients are to be found who might benefit from a fresh appraisal.

Once a patient has acquired a label in one's mind it is easy to accept implications that are not always justifiable. A patient with hemiplegia, especially if this is accompanied by dysarthria or aphasia, may suffer through ignorance of the possibilities of rehabilitation, or just from lack of a clinical review from an unjustified degree of incapacity. Twice recently I have admitted such patients to relieve strain on their family supports and have found that a very material improvement in mobility and aptitude could be achieved when a proper attempt was made, to the great benefit of both patient and family. If the possibility of rehabilitation is constantly borne in mind surprising results may be achieved. Some people become vastly more efficient if they can use hearing aids or if ophthalmic treatment is possible. It is incumbent on *someone* in each case to think of the possibility.

Senile dementia is a very dangerous label for it denies all hope of recovery. Beware of it for it is not such a very common condition. Confused states of mind can, as we all know, derive from such diverse states as chemical imbalance (hypoglycaemia or uraemia), anoxia due to anaemia, carotid and cerebral atherosclerosis or congestive cardiac failure, as well as from personality withdrawal and frank psychosis. Many, perhaps most confusional states are reversible. I have seen more than one so-called senile dementia recover after a course of enemata.

Again if the term exists in your vocabulary strike out "myocardial degeneration". As a clinical term it means almost nothing but ischaemic heart disease with widespread fibrosis. Banish the vague chimera of a senile degenerate myocardium from the mind of the profession and many more cases of malnutrition, dehydration, anaemia and bronchopneumonia will be diagnosed and treated. In frank cardiac disease the clinical concept of myocardial degeneration specific to ageing seems to carry the implication that treatment will fail, whereas it is the case that cardiac failure and disease in old people are no less responsive to treatment than in middle life. Normal heart muscle would seem to be almost immortal!

Cardiac failure must always be regarded as a symptom, not a diagnosis. I have for instance recently had two cases of dropsical failure which responded to opposite approaches. One man with tachycardia and high output failure, warm blue hands and a history of wandering round his village in a strange and frightening way was found to be in a thyrotoxic state though the thyroid gland was not enlarged. When treated with carbimazole he recovered completely. A woman with equally severe dropsy was so tired and lethargic she had no wish to live—the coarse skin, hoarse voice and bitter complaint of the cold weather made the diagnosis of myxoedema obvious, and she recovered well on thyroid treatment. In old age thyroid dysfunction is often atypical in presentation but if one keeps the possibility in mind myxoedema, which is fairly common, will seldom be missed; thyrotoxicosis being quite rare may be less easy to spot.

Coronary thrombosis in old age usually presents without significant pain. It may be that failing memory makes the patient a bad historian, or perhaps it may be due to the increasing collateral system of arteries which develops in late years. Whatever the cause severe cardiac pain is a rare complaint. The unexpected onset of congestive failure, a sudden faintness, even a fall, may be the only indication. Recently a woman aged 68, a known diabetic, came under my care having collapsed the day before and begun to vomit. Vomiting continued for several hours and she was admitted in diabetic coma precipitated by vomiting due to myocardial infarction.

In diagnosis *ab initio* and in continued care we must remember that multiple pathology is almost the rule in old age. More and more often it has been borne in upon me how close an eye must be kept on geriatric patients in the acute assessment ward. Many of them pass through a series of disease states any one of which might carry them off. Whatever the presenting diagnosis there is a risk of bed sores, pneumonia, urinary infection and thrombophlebitis occurring. Till I began to practise geriatrics I never dreamed how common fatal pulmonary infarction could be. Recurrent anaemia due to intestinal haemorrhage is astonishingly common, usually without a demonstrable source. One must keep sharply alert in these wards. Time can help us only if we know what we are up against. Life is certainly easier in acute wards where so many of the patients make one diagnosis do for one admission! The risk of embolic phenomena is much in my mind especially when stroke occurs in the presence of auricular fibrillation. Arterial emboli of every degree are ever present possibilities in such cases and justify to a late age the use of anti-coagulant drugs in cerebral embolism and in myocardial infarction.

Sometimes disease states which have previously been present in mild degree achieve prominence because of social crises. A short time ago I was asked to see an old woman who had taken to her bed with pneumonia and generalized senile tremor. A visit to the house unmasked a severe case of iron deficiency anaemia in the aged husband. This had remained unnoticed because while the wife was able to continue her fairly active life the husband had just sat still most of the day.

In another instance a pair of old ladies lived together, one in effect looking after the other. When the prop came into hospital with a myocardial infarct the other came in as a social necessity and was found to have a profound degree of pernicious anaemia. At the time of their discharge the orginal status of the two women was to a large extent reversed. The dependent one had become physically vigorous whereas the original prop was still in mental outlook an invalid.

In a third case a cerebral thrombosis of minor degree, which incapacitated a vigorous healthy man of 82, threw an intolerable strain on his wife's cardiac reserves and admission of both parties to hospital became an urgent necessity. As is so often the case the man recovered well, long before his wife whose breakdown disclosed a state of fatigue of long standing, congestive cardiac failure and troublesome cervical spondylosis.

### REHABILITATION

The third phase of medicine (Rusk, 1959) consists of re-training the patient to the needs of daily life and restoring to normal function the disabled limbs. In old people this can be a difficult, protracted, and often disappointing business, but whenever it can be it should be done. It is easy to say that all the old folks want is to be allowed to rest after their labours, but prolonged bed rest carries very real dangers. Even the period of 6 to 8 weeks immobilization which was once required for fractures of the neck of the femur carries a risk of rapid decalcification of the skeleton, loss of muscle power, of bladder control, even of reason itself, so that this treatment has been described as hanging the patient by the leg till she is dead! (Crockett, 1960). Geriatric physicians live in dread of these consequences of bed rest. Osteoporosis indeed is so common a condition as almost to be a normal accompaniment of old age in women. Any disease process or any treatment involving prolonged bed rest can accelerate its insidious progress and bring about disaster, so it is not surprising that the bedrock of treatment and rehabilitation in the elderly is to get the patient up and dressed. If we feel like human beings we are more likely to behave as such. If for 80 years we have been accustomed to certain standards, preservation of as much normality as possible in daily life will help to maintain *amour propre* and even to restore reason to the confused mind. The normal position of human beings is upright. If the old are confined to bed many of them withdraw from reality and sink back into infantile dependence.

Rehabilitation takes many forms. Care of the feet is of prime importance. One cannot walk on painful feet with any ease. Chiropody should be readily available and I personally make it a practice to conduct physical examination from the feet upwards. If the feet are sound and if suitable footwear or ankle supports are selected it is surprising what obstacles of skeletal deformity or neurological defect can be overcome, provided unnecessary strain is avoided, and in this context I must remind you that obesity is a terrible handicap in old age. The idea of carrying 20, 30, or 50 extra pounds about all day long is one which nobody would entertain if the weight was other than bodily fat. As physical strength decreases gravity may at last become the dominant force. Provision for ageing should take account of this danger.

I should like to illustrate our general attitude to rehabilitation by reference to hemiplegia. It is true for practical purposes that recovery of the arm and hand begins within 3 months or not at all. If little or no recovery occurs in this period it is wise to concentrate effort on teaching the patient to use the other hand comprehensively.

The paralytic leg on the contrary may become a useful member later than this and we do not despair of recovery till 6 months or more have passed. From the earliest possible stage we encourage the maintenance of muscle tone in the quadriceps femoris, and by passive movements of arm and leg seek to preserve the full range of joint movement. Everything possible must be done to prevent "freezing" up, of the shoulder especially, so that such muscular recovery as does occur will not be unnecessarily embarrassed. Usually the leg does better than the arm. Perhaps most such cases can learn to walk with the help of a stick or a tripod if the cerebral cortex and personality remain sufficiently intact. When there is no recovery of the leg it can usually still be of some value as a spastic support, but in these cases we must hope for recovery of the arm so that two tripod sticks or a light walking aid, or even crutches, will make mobility possible. In short, one must in all cases make the most of such function as can be preserved.

Aphasia may be a most disabling consequence of cerebral thrombosis and hemianaesthesia makes rehabilitation more difficult. The situation is still worse if homonymous hemianopia is present. But in such cases the dominant factors are first the patient's personality (this is all important), and second the enthusiasm and persistence of the team which has to be maintained despite repeated disappointment. My own attitude, which is I think tempered by a reasonable clinical common sense, is that any degree of independence is worth seeking. If the patient can do no more than feed himself and write letters, that is really valuable to some people; but if he can walk, however slowly, he can escape the most profound humiliations.

### INTEGRATION OF SERVICES

Care of the elderly is divided between the general practitioner, the hospitals and Local Authority. Once a state of failing independence is reached we must consider whether more help is needed than the family can provide. The Local Authority is responsible for maintaining Welfare Homes, Health Visitors, the Home Help Service, District Nursing, Meals on Wheels, Chiropody, etc. The hospital Board is officially responsible only for those who need hospital services. In rare instances the geriatric physician is also physician to the Welfare Homes and in such situations traffic between Homes and Hospitals moves with great freedom—but this is a very exceptional arrangement and increasingly it is the vogue to integrate the Geriatric Hospital Service with that of general medicine. I think this is correct; they are different aspects of the same thing. Nevertheless the geriatric physician should be able to guide both the Welfare Department and the general practitioner so that one provides what the other needs and both know what services can be made available to people in need.

How far is the Geriatric Service from being a success? I think that few places indeed can claim that they have a wholly satisfactory service, and certainly in this clinical area it would be surprising if this were the case. A unified service was only initiated 7 months ago and we are a long way from reaching our peak of efficiency. It probably is true that a considerable number of patients became "chronics" in the past who today might be maintained at a reasonable level of independence. But they still occupy hospital beds and till they die those beds remain occupied. Gradually however an increase in the speed of turnover and hence in the number of patients admitted and discharged is becoming perceptible. In the last 6 months 501 patients were admitted to the hospitals of my service, compared with 413 in the corresponding period last year. Moreover the relation between death and discharge as the probable consequence of admission has been reversed.

| | | | |
|---|---|---|---|
| Deaths, | January—July | 1959 | 216 (52 per cent) |
| ,, | ,, | 1960 | 227 (45 per cent) |

More important perhaps to the general practitioner is the fact that there are fewer patients on the waiting list than there used to be and these get into hospital quicker.

In the last 4 months no one has had to wait as long as 4 weeks for a bed and many have been taken in within days of request.

The future value of the service however must lie not in coping with the urgent needs expressed by general practitioners' demands for hospital beds, but in an expansion of the service directed at prevention of decay. Sometimes it is now possible by joint consultation between the general practitioner and geriatric physician so to deploy resources as to maintain people in their homes without admission or with only brief periods of in-patient treatment. Expansion of the Domiciliary Welfare Services will help in this direction. Meals on Wheels is one such service which should be expanded. Friendly, informal visiting, or regular transport to an Old People's Club may make the difference between normal mental alertness and the insiduous onset of personality withdrawal and dementia. Occupational therapy at home may sometimes improve a domestic situation which without it would be intolerable.

A vigorous out-patient service probably can be of real value both to the general practitioner and to the discharged patient. In Oxford especially a Day Hospital which patients attend one, two, or three days each week makes long term rehabilitation possible through occupational therapy and provides much needed relief for the relatives especially of confused patients. We cannot aspire to such luxury as Oxford has but we have started a Day Hospital in a small way in Cheltenham, and such things grow.

Close co-operation with the psychiatrists is mutually valuable. We are setting up a workable arrangement whereby both they and I can admit cases of severe confusion to certain beds in which my psychiatric colleagues and I will examine and assess each patient and ensure that the most suitable treatment and disposal is arranged.

We have a long way to go to reach the standard I seek and time is not on our side—the numbers of old people grow. But I am very sure of one important point. The solution to the problem does not lie in adding more and more chronic sick beds where chronic neglect is the medical routine. It lies rather in the vigorous use of well equipped beds by interested medical men. Geriatrics is not a specialty; it is a technique, and above all it is an attitude of mind which will one day be taught to the medical student and will become an integral part of the attitude of every consultant and every practitioner in general medicine.

### REFERENCES

Crockett, G. S. (1960). *Brit. J. Clin. Prac.*, **14**, 385.

DeLargy, J. (1957). *Lancet*, *i*, 418.

Rusk, H. A. (1959). *Practitioner*, **183**, 505.

Isaacs, B. and Thompson, J. (1960) *Lancet*, *i*, 969.

## Figures and Tables

**Figure f1:**